# On Standardized Measurement in Behavioral Science

**DOI:** 10.17505/jpor.2022.24854

**Published:** 2022-12-22

**Authors:** John R. Nesselroade, Peter C. M. Molenaar

**Affiliations:** 1 The University of Virginia emeritus; 2 The Pennsylvania State University emeritus

**Keywords:** idiographic filter, measurement, P-technique, intraindividual variability

## Abstract

That standardized measurement procedures are a sine qua non of “good” science is generally not questioned. Here we examine the meaning and use of standardized measurement in behavioral science. Procedures and methods of measurement that have served the physical sciences so well should not blindly be assumed to work in the same manner and with the same effectiveness in behavioral science. There seems to be general agreement that social/behavioral science is “different” among the sciences. Problems arising from how behavioral science is “different” begin, we believe, with measurement. We put forward the argument that the source of the difference is unique to animate objects and is first evident at the stage of measuring the behavioral attributes of interest. It is at that point in conducting scientific inquiry that the matters raised might be resolved by developing and applying alternatives to standardized measurement. One such alternative discussed is the idiographic filter (Nesselroade, Gerstorf, Hardy, & Ram, 2007).

Behavioral science has strived mightily to emulate physics and other “hard” sciences by adopting their appreciation for rigor as well as many of their methods. In this discussion, we want to focus on one particular methodological innovation – standardized measurement – because we believe it to be a major culprit interfering with behavioral science’s further advance. We contend that a critical evaluation regarding early behavioral science’s adoption of standardized measurement procedures, so useful in the physical sciences, is in order and that a thoughtful reappraisal of standardized measurement sets the stage for adopting other innovations in behavioral measurement.

The concept of *standardized measurement* (not to be confused with standardized scores, e.g., *z*-scores) has great credence in science. But might not the concept be wrong, or at least misapplied, in the case of behavioral science? Elsewhere, we (Nesselroade & Molenaar, 2016) argued that the slavish application of traditional measurement thinking to the study of living beings’ behavior, rather than the answer to the question of how to study behavior scientifically, could be a detriment to such pursuits. We recognized that behavioral science differs in important ways from physical science but proposed that the core of the difference between them did not directly have to do with the question of whether or not humans could rigorously study humans but that the proper measurement of behavioral attributes of animate objects might have to be done differently than the measurement of attributes of inanimate ones. Now, we wish to take our arguments further to suggest that the real culprit is the use of standardized measurement protocols on animate entities.

With regard to differences in animate versus inanimate objects, Greene (2020) noted that both rocks and rabbits can exhibit movement but a rock’s movements are accounted for completely by external forces whereas a rabbit’s movements are governed in part by internal events. That is, a rock’s movement is completely predictable from knowledge of the external forces acting on it. A rabbit’s movement is only partially predictable from knowledge of the external forces acting on it (e.g., the barking of a dog). Internal forces (e.g., hunger) also help determine a rabbit’s movement. This difference bears heavily on the appropriate use of standardized measurement. Conventional standardized measurement is designed to cope with the external forces and not the internal ones. This makes it appropriate to apply standardized measurement protocols to inanimate objects but not necessarily to animate ones.

## Standardized Measurement

The value, nature, and techniques of standardized measurement have been richly discussed in the behavioral science literature (e. g., Guilford, 1954; Gulliksen, 1950; Nunnally, 1967). Consider a “common sense” notion of standardized measurement: “*Standardization - All procedures and steps must be conducted with consistency and under the same environment to achieve the same testing performance from those being tested.*” (wikipedia.org/wiki/Psychological testing)

The purpose of standardizing measurement is to ensure that the values obtained can be meaningfully compared. Whether they are scores for different entities measured at the same time or scores for the same entity measured at different times, meaningful conclusions drawn from comparing two scores rest on the scores being obtained in such a way that the very act of obtaining them did not influence their values differentially. If two scores are different, it should be because the amounts of the attribute being assessed differ; not because the difference was introduced by the act of measurement. Typically, great care is taken to guarantee that scores obtained in a measurement procedure have these comparability features. For example, testing conditions and procedures are strictly controlled in an effort to provide uniformity in the meaning of the resulting numbers.

We understand “to achieve the same testing performance” to mean not the same score, necessarily, but that the scores assigned to different individuals are measurements of the same thing. More pointedly, what does it mean to conduct the procedures and steps “under the same environment?” With regard to behavior, it is our contention that it is the focal performance being measured that needs to be in the “same environment” and not merely the physical vehicle (person) doing the focal performance. In addition to room lighting, time of day, ambient temperature, length of time allowed, verbal instructions, etc., (in Greene’s terms the external forces) the environment for the focal performance also includes what else the individual brings to the measurement event (i.e., his or her levels of understanding, interest, motivation, expectations, competitiveness, etc., (in Greene’s terms the internal forces) that impinges on the focal performance.

Regarding measuring movement in rocks and rabbits referred to earlier, external forces, conditions, etc., are proper foci for standardized measurement but internal events may be beyond reach. Consider something as apparently straightforward as squeezing a dynamometer. Certainly, external forces (resistance, ambient temperature, humidity, lighting, etc.) can be controlled so that differences in performance reflect differences in physical strength (standardized measurement). But differences in performance also reflect differences in motivation, interest, competitiveness, boredom, etc., which are not the same from one individual to the next (unstandardized measurement). Instructing participants to squeeze as hard as they possibly can does not equate different individuals on all the influential attributes that are in play, in addition to physical grip strength. And therein lies the rub. Without such equating, how can we call the scores obtained by different participants the products of standardized measurement, thus rendering them comparable and worthy of being cast into the same score distribution for further analysis?

Univariate/bivariate research designs recognize the multiple impinging influences on behavior and try to control for their influence with devices such as random assignment to experimental conditions and bringing participants to some performance criterion prior to assessment. But such “fixes” are aimed at average group performances rather than individual ones. Regard for the multiple influences on a given focal performance accounts for some of the appeal of multivariate approaches to the study of behavior (Cattell, 1966b), which has helped lead to greater use of latent variables (e.g., factors) as a path to accounting for the multiple influences underlying observable performance.

Once the considerations just raised are admitted, being able to provide the same environment required for behavioral measurements to represent a common referent (to be truly standardized) seems, for many situations, a forlorn hope. The foregoing concerns can (and should) be raised regarding repeated measurements of the same individual. It may be Mary Smith on two different measurement occasions, but there is no guarantee that Mary is bringing the same pattern of impinging attributes to the focal performance. Thus, an individual may not be a worthy “control” for himself/herself in a repeated measurement situation.

For the measurement of behavioral attributes to be truly standardized, internal forces acting on the participants must be accounted for (controlled) just as the external forces acting on participants must be. Occasionally, meager attempts at doing this are made as when some measures are used as covariates in an ANOVA design but this cannot be considered a substitute for standardized measurement. Before identifying one alternate approach to measurement to deal with the matters we are raising, we will lead up to it by briefly considering three ideas that are evident, if not universally embraced, in the literature of behavioral science. These are familiar topics but they are essential to our purpose and thus warrant brief additional discussion The first is the recognition of the individual as the proper unit of analysis in studying behavior. The second is the value of working explicitly with unobserved (latent) variables and their observed indicators – manifest variables. The third is the role of invariant relations in empirically-based inquiry into human behavior.

## The Individual as the Unit of Analysis

The individual is the primary unit of analysis for studying behavior, not the differences among individuals which are featured by the differential approach. This notion is by no means new (see e.g., Carlson, 1971; Magnusson, 2000; Molenaar, 2004) but psychology’s huge investment in studying individual differences has helped delay its general acceptance. But there are signs that the tide may be turning. For example, in medicine, a rapidly growing emphasis on personalized diagnoses and treatment regimens reflects a renewed emphasis on focusing on the individual. Somewhat ironically, perhaps, this emphasis on individuality has been accelerated by advances in genetics, a discipline that has in many ways nurtured the differential approach. Adaptive testing is another example of personalized attention to individuals when conducting measurements. But adaptive testing is based on models of between-subjects variation which rest on the notion of standardized measurement. The roots of the emphasis on individual differences have been critically examined by Molenaar (2004).

Another of the main directions in which the study of behavior from an individual orientation has headed is a more general and rigorous focus on the study of processes in contrast to more or less static attributes. An emphasis on the individual rather than on individual differences as the unit of analysis is highly pertinent to the study of process. Processes happen over time so studying them requires a more extended collection of information about a given individual. As we have illustrated elsewhere (Molenaar & Nesselroade, 2012) a process can be rigorously identified as a general conception at the latent level with its particular observed manifestations being specific to a given individual. Such an idiosyncratic notion of process is not congruent with the conception of standardized measurement but its fit to empirical data can be rigorously evaluated statistically and rejected if the data so dictate (Molenaar & Nesselroade, 2012).^1^

It is key to highlight the distinction between latent and manifest variables as we do in the next section and in subsequent discussion. Here and elsewhere, our general orientation is to try to keep individuality out of the relations among latent variables and let it appear in the manifest variables and their relations to the latent variables, as we discuss later. For the study of process this separation of individuality from general lawfulness has the promise of being able to identify process at the latent level for the individual while showing it to be general to other individuals. The idiosyncratic aspects of the general process (how it manifests) at the observable level can be identified and separated from the latent process for different individuals. But if generality resides at the latent, rather than the manifest level, it will not be detected at the manifest level, in which case a strong focus on standardized measurement (the manifest level) represents the road to failure.

Of some interest in regard to the individual as unit of analysis is the matter of predicting behavior. Prediction of behavior within the differential framework relies heavily on the differences between individuals. Measured differences between individuals’ attributes are used to predict differences in outcomes. Putting a strong emphasis on the individual as the unit of analysis rather than emphasizing differences among individuals invites building prediction schemes in other ways. One obvious possibility is to use the individual’s past behavior to predict his/her current or future behavior. Though at odds with differential psychology’s main approach to prediction, the use of past behavior of the individual (e.g., reinforcement history) to predict current behavior is obviously congruent with some forms of learning theory.

It seems that there are many considerations that point to the importance of explicitly recognizing the individual as the proper unit of analysis. If one accepts this idea, then we should ensure that the act of measurement of behavioral attributes be conducted in a way that does not negate it. Treating different people’s scores that derive from what are thought to be standardized measurement protocols, but aren’t for reasons suggested above, is not a useful way to keep the individual the unit of analysis. It seems far more appropriate to admit that, from a traditional standardized measurement perspective, between–person comparisons on the manifest variable level might not be meaningful. In that case, the aggregation of data over different individuals is problematic.

## Latent Variable Modeling

Latent variables are central to psychological research and theorizing. They populate our research and theory, requiring the development of measurement models (Borsboom et al., 2003; Jöreskog & Goldberger, 1975; Meredith, 1993; Millsap & Meredith, 2007). The structural equation modeling effort has advanced the rigorous use of latent variable modeling through its emphasis on developing “convincing” measurement models for latent variables. Generally, some version of a multivariate analysis approach is used to build and test the measurement models.

As to the impact of errors of measurement when focusing on individuals rather than groups, modeling with latent variables offers ways to split off measurement error when modeling individuals. Considering psychometric concepts such as reliability at the individual level (Hu et al., 2016) provides much more nuanced information about the character of the measurements than do group-based statistics.

Different fields of inquiry have developed their general principles using concepts and interrelations defined at the latent level. The geometric relation between the area and radius of a circle, A = π r^2^, works for any circle made out of any material, at the equator or at the North or South poles.^2^

It is not a huge step from a multivariate orientation to the incorporation of latent variables into one’s thinking. Previously (Nesselroade & Molenaar, 2016), we discussed how latent variable modeling seems to provide one way to avoid a strict adherence to the practice of standardized measurement while still supporting the measurement efforts needed to lead to the establishment of lawful relations in behavior. More will be said later about this notion, termed the *idiographic filter*.

Relations among latent variables provide the generality that lawful relations need to be scientifically useful. For example, “Crushing large stones with a sledge hammer gives one a backache” does not have the same pertinence to one who never uses a hammer as it has for someone who does. Whereas “Hard manual labor produces muscle fatigue” has much greater generality; it holds for the hammer wielder but also for one hoeing corn or pushing a lawn mower; behaviors affecting different muscular configurations.

But latent variables are not directly observed. Their properties must be inferred from manifest variables that serve as the latent variable’s indicators. Establishing the linkages between manifest and latent variables is a primary focus of measurement (Borsboom et al., 2003; Meredith, 1993; Nesselroade & Molenaar, 2016), but there we began calling into question the practices of standardized measurement by arguing that a measurement scheme may have to be different for different individuals in order to access the same latent variables.

Nesselroade and Molenaar (2016) identified a number of areas in which there is evidence to suggest that particular manifest variables do not provide a “foolproof” link to the latent variables they are supposed to index. Included were self-report, learning task performance, specificity of response in autonomic nervous system activity, and brain imaging. The diversity of areas suggests a very broad applicability of the concerns raised in our critique of behavioral measurement practices. These examples emphasize the point that an observed variable may not functionally be the same manifest variable from one individual to another and therefore is not appropriate for aggregating information across individuals. A clear example used before concerns the adjective “anxious.” When two people rate themselves as “anxious,” one may be thinking about anxiety while another is thinking about eagerness.

To ask “How well do you play tennis?” may generate answers from players and non-players of tennis but the responses are hardly comparable. Nevertheless, this would be deemed a standardized measurement compared to asking “How well do you play your favorite sport?” which can generate comparable answers from both tennis and non-tennis players. Tennis is manifest; favorite sport is latent and only derives its specificity by considering the individual. Trying to mitigate the problem by restricting the study population to homogeneous sub-populations isn’t apt to work because homogeneous sub-populations are very difficult to identify a priori due to the extent of subject-specificity.

The point in geometry has much in common with the concept of a latent variable. Consider the following. A point, usually represented by a dot, has no dimensionality: no length, no width, no height. Therefore, a point cannot be observed. It can be talked about as though it exists because it has a location; a location typically specified by coordinates on some reference system. Latent variables, similarly, cannot be directly observed. They are indexed by manifest variables that can be observed. We can talk about latent variables, even their relations to other latent variables in the abstract, just as we talk about points in space, but their reality is given through their manifest indicators – their coordinates in the case of points. But, in order to do something with it – to manipulate it – a point has to be indexed by its coordinates such as x and y in a two-dimensional Cartesian space. Consider the distance between two points on a plane. The Cartesian coordinates x_1_, y_1_ and x_2_, y_2_ are observable indicators of hypothetical points p_1_ and p_2_. By using x_1_, y_1_ and x_2_, y_2_ one can calculate the distance between p_1_ and p_2_ even though the two points cannot be observed.

To fully apprehend the significance of our concerns regarding latent variable modeling and standardized measurement, we must rely on the concept of invariant relations and their central role in science, the topic to which we now turn.

## The Role of Invariant Relations

Keyser (1956) emphasized the importance in science of ascertaining which attributes of objects remain invariant under which transformations. This is a very general statement but one which is fundamental and highly germane to behavioral science. If identifying and using invariant relations is a key component of developing scientific knowledge, then behavioral science should be focusing on the matter directly. While it is the case that measurement in behavioral science has often emphasized demonstrating invariant relations, for instance those between a collection of manifest variables and the latent variable they index, the slavish use of standardized measurement procedures has figured prominently in attempts to establish these invariant relations and our critique has raised the possibility that it is not appropriate.

From the standpoint of what standardized measurement purports to accomplish, there are two distinct matters to consider. The first is to ensure that the scores obtained for different individuals represent a common referent. That is to say, that the meaning of the scores is invariant from person to person or from occasion to occasion for the same person so that the scores of different people or obtained from an individual at different times can be meaningfully compared. As noted at the beginning, this assurance is supposedly provided by using standardized measurement procedures – an expectation that we are seriously questioning.

The second matter has to do with the establishment of invariant relations between obtained scores and the latent variables they purport to index. This situation, referred to as *measurement invariance* (see e.g., Millsap, 1995), has been called into question (Nesselroade, 2007; Nesselroade & Molenaar, 2016) on the basis that if the first matter is not resolved appropriately by the act of standardized measurement, i.e., if standardized measurement does not guarantee that the meaning of the scores remains invariant from one individual to another, it raises doubts regarding the second matter. The idiographic filter proposes, instead, that the invariant relations we seek as behavioral scientists are to be found among the latent variables, and the array of manifest variables indexing those latent variables might have to be different from one individual to another. The fit to empirical data of this measurement approach can be assessed statistically for both static attributes and processes (Zhang et al., 2011; Molenaar & Nesselroade, 2012) and rejected as untenable if empirical data so ordain.

Nesselroade et al. (2007) illustrated the notion of invariant relations among abstractions such as latent variables when the observed variables differ with this geometric example: Consider cylinders, cuboids, and triangular prisms. Each has a cross-sectional area, A, that rests on different observable features: length and width, the radius (squared), and altitude times base (halved), respectively. Each also has a volume, V. For all three objects, V = A · s, where s is the physical length of the object. Thus, the relation between the abstract properties (A and V) is invariant over the three kinds of solids, even though A in each case rests on different measurable attributes.

Consider another example of invariant relations among abstractions, this one from physics. The momentum of a moving object is generally defined as the product of its mass times its velocity. This definition applies to an object moving in a straight line. Rotating/revolving objects also have momentum. In this case, (angular) momentum is also defined as mass times velocity but mass is now the *moment of inertia* (how much mass and how it is distributed) and velocity is angular velocity (the speed at which the body is turning). Thus, in the case of linear versus angular momentum, the factors that make up the mass and the velocity are measured differently but the abstract relation, that momentum is the product of mass times velocity, is invariant over the two kinds of systems.

In behavioral science measurement, we often don’t think in terms of the important invariant relations being between latent variables. Instead, the invariance focus is often on the relations between a given latent variable and its observed indicators (measurement invariance) or between manifest variables. The relations among latent variables are often considered to be matters for empirical discovery. However, in the examples just given (e.g., momentum, mass, and velocity) it is the relations between latent variables that allow for general statements of invariance (read lawful relations).

Invariant relations among latent variables satisfies the scientific need for attributes that remain invariant under different transformations. Harking back to Keyser’s comment, using different configurations of manifest variables represents different transformations and invariant relations among latent variables is a key kind of invariance. It is noteworthy that in the examples given, these invariant relations are identified without using traditional standardized measurement protocols. Although perhaps not mechanisms themselves, invariant relations among latent variables are possibly the ”footprints” of mechanisms, processes, etc., as they are portrayed by the constraining structure of a correlation or covariation matrix.

As pointed out by Nesselroade et al. (2007) invariant relations among latent variables guarantee that there will be invariant relations between them and the more abstract latent variables (e.g., second-order factors) derived from those invariant relations. This is a more abstract form of measurement invariance (higher-order invariance) that may provide an avenue to general lawful relations (mechanisms, processes) even if the empirical referents – the manifest variables with which we index and test lawful relations – differ.

In relation to the earlier discussion of idiosyncratically different indicators for latent variables, the two hypothetical points p_1_ and p_2_ could also have as observable indicators their polar coordinates r_1_, *θ_1_* and r_2_, *θ_2_* instead of Cartesian coordinates. For the two cases, the observables are measured quite differently but the distance between the points (p_1_ and p_2_) remains invariant under the transformation from Cartesian coordinates to polar coordinates and vice versa.

The forgoing arguments have been aimed at weakening, if not demolishing, the case for standardized measurement protocols in behavioral research, mainly because they may not work. With different arrays of manifest variables for different persons to index the same latent variables the traditional notion of standardized measurement becomes irrelevant. But note that we are not giving up the objective of identifying lawful relations among concepts that are invariant over individuals. Nor are we arguing for the abandonment of empirical research for testing proposed relations among concepts. Rather, we are arguing for recognizing that the goal is the establishment of invariant lawful relations among abstractions just as for any other scientific discipline. We do not think, however, that this goal is attainable through the use of standardized measurement protocols.

## Multivariate, Replicated Single-subject Repeated Measurements Designs (MRSRM Design)

“The possibility that a given observable may have a different significance for different persons, or that different observables may signify the same thing to different persons raises doubts about the mindless aggregation of data in group designs. The distinction between observable behaviors and inferred constructs… provides a conceptually powerful way to address the matter of replicability either at the level of observables… or at the level of unobservables inferred from multivariate covariation patterns. (Nesselroade & Ford, 1985, p. 67).

Nesselroade and Ford (1985) were arguing for the value of what Cattell (1952) labeled P-technique data, with its emphasis on repeated multivariate measurements of the individual. They examined the merits of designing empirical studies around a collection of individuals so measured to identify patterns of intraindividual variability and their replicability across individuals. They termed the configuration Multivariate, Replicated Single-subject Repeated Measurements Designs (MRSRM Designs).

**Figure 1:**
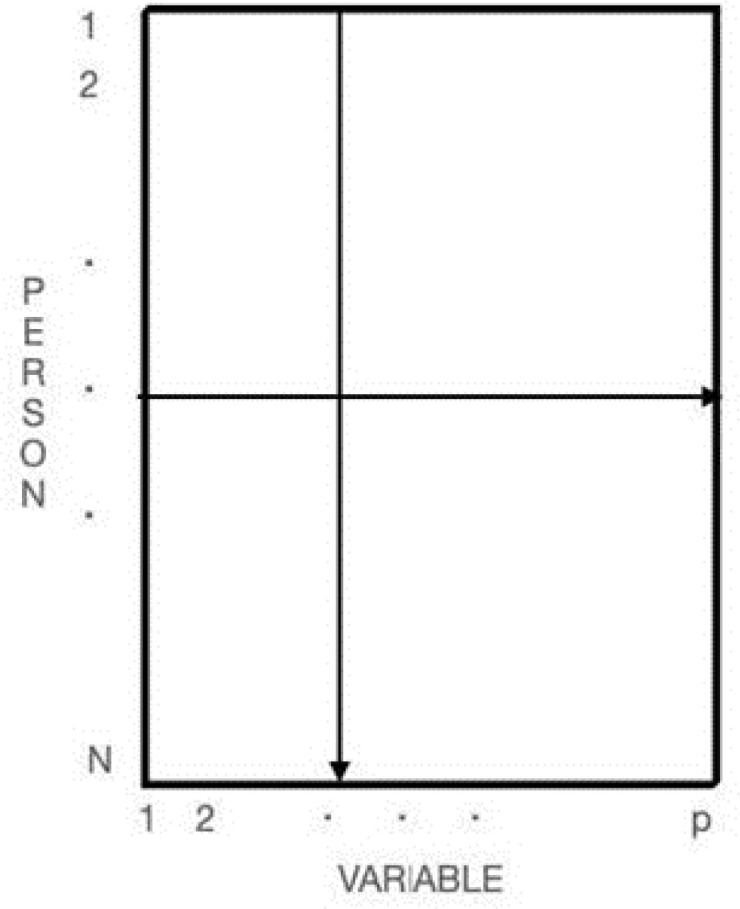
General N (persons) by p (variables) Data Matrix

To further illustrate the implications of our concerns regarding standardized measurement and its possible shortcomings, we will first construct and then deconstruct the three-dimensional data box popularized by Cattell (1966a). First, consider an ordinary two-dimensional *N* persons × *p* variables data matrix as shown in Figure 1.

**Figure 2:**
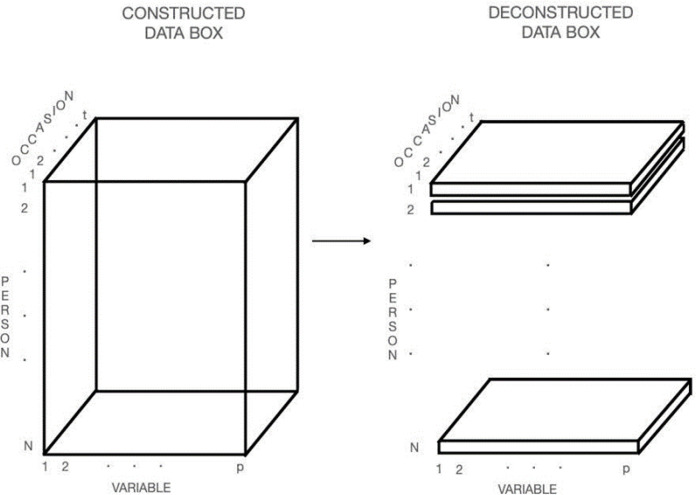
Construction and Deconstruction of the Data Box

This *N* × *p* data matrix in Figure 1 can be elaborated into the three-dimensional “data box” by adding *t* additional *N* × *p* “slices” to represent a succession of *t* occasions of repeated measurement as shown on the left side of Figure 2.

To illustrate the possibility that relations between observed and latent variables are subject–specific, even though the former may bear the same label, we uncouple the *N* “slices” of Variables × Occasions data. The result, shown on the right side of Figure 2, is a collection of data matrices, one for each person, that corresponds to the series of P-technique data matrices that Nesselroade and Ford (1985) called the MRSRM Design. Thus, this design explicitly recognizes that observed and latent variable relations may be subject-specific, thereby emphasizing the individual as the primary unit of analysis.

## The Idiographic Filter

What seems clear is that it is not some abstract notion of standardized measurement *per se* that is found lacking; rather what is problematic is the way it has been utilized in studying behavior. Behavioral science has generally assumed that the mappings of latent onto manifest variables are the same for individuals which is a key premise for the use of standardized measurement and for the unquestioning aggregation of data across individuals. How else can one justify organizing data in a persons by variables rectangular data matrix as the basis for ascertaining patterns of relations?

As mentioned earlier, the fundamental idea underlying the idiographic filter is that the invariant relations that are so prized in scientific research and theory development reside at the level of relations among latent variables, not among manifest variables or between manifest and latent variables. Here, and elsewhere (Nesselroade & Molenaar, 2016) we have raised the possibility that “cornering” these latent variables may not be possible with a battery of manifest variables fixed to be the same across individuals. Obviously, this is antithetical to a straightforward notion of standardized measurement. Emphasizing invariance of relations among latent variables has the advantages of allowing the researcher to employ idiosyncratic measurement schemes while maintaining a rigorous version of the invariant relations that are involved in general lawfulness.

The Idiographic filter emphasizes the latent variables and their interrelations over the manifest variables and their interrelations. The former are embedded in a system of relations that can be (and are) tested for invariance across individuals using standard model fitting tests. Thus the latent variables derive their meaning from the system of relations with other latent variables as well as relations to manifest variables. Given invariant relations among the latent variables over individuals, the richer the web of latent variables is the more confidence one can have in the identification of the latent variables. As more experience with fitting the idiographic filter models to data accrues, our understanding of the full meaning of the person as the primary unit of analysis will also become clearer.

Obviously, there are many tasks attendant to the measurement approach we are advocating here (e.g., how to scale latent variable scores when different manifest variable configurations are used for different individuals.) We cannot resolve such matters here but we hope that the case we are trying to make for a different way of thinking about standardized measurement in studying behavioral attributes will coax others to work on appropriate solutions.

For the empirically minded, locating invariant relations at the latent level may at first seem problematic, but it is not. Mathematical transformations (Cattell, 1966c; Schmidt & Leiman, 1957) exist that project these higher-order latent variables onto the manifest variables, giving the higher-order latent variables a tie-in to the variables that can actually be measured. Thus, there is a bridge from the invariant relations in latent space to the manifest variables. But the case may be that the mapping of latent variables onto manifest variables may be somewhat unique to the individual. Or, possibly even unique to the individual at a given point in time. Doubtless, these individual mappings may show a great deal of similarity and, in special cases be the same, but that is an empirical matter to be examined. If consistency across individuals emerges in the context of allowing idiosyncrasy, so much the better.

## An Example

Elsewhere, an application of the idiographic filter to empirical data has been presented in detail (Nesselroade et al., 2007). The example involves the self-ratings on an affect adjective checklist by five pregnant women, completed daily by each participant for more than 100 occasions of measurement (Lebo & Nesselroade, 1978). Each of the five participant’s string of measurements included the day their child was delivered. The data represent the MRSRM design described earlier i.e., 5 P-techniques.

The objective of the factor analyses of these data was to identify expected affect dimensions based on the selection of adjectives used in constructing the checklist and assess their consistency across the five participants. The data were modeled in two essential ways. First, a more standard “measurement invariance” approach in which factor loading patterns were constrained to be invariant over participants and factor covariance matrices were free to vary was conducted. Second, the idiographic filter was implemented by constraining the factor covariance matrices to be invariant across participants with the factor loading patterns allowed to vary idiosyncratically.

Model fit indices leant support to the idiographic filter as providing the better fit to the data. There are two main points stemming from this conclusion. The first is that because the factor covariance matrices are invariant across participants, a higher-order factor analysis (e.g., second-order analysis) would, of necessity, result in factor loading patterns that are invariant across participants. Indeed this was tried and the two resulting invariant second-order factors were interpretable as activity and affect. Admittedly, second-order factors are abstract, but that is the point of the idiographic filter notion. Invariant relations are going to be found at an abstract level, not at the level of manifest variable/construct relations. The second point is that despite the first-order factor loading patterns not being invariant, they were still similar enough over participants and to the hypothesized factors based on the selection of adjectives to warrant the interpretation of idiosyncratic manifest features of the same underlying constructs. These findings leant substantial support to the idiographic filter conception of expecting invariant relations at the level of the constructs rather than at the level of relations between manifest variables and constructs.

## Whither Standardized Measurement?

We have argued for a reassessment of the concept of standardized measurement and its implementation in behavioral science. Our primary argument is that multiple forces, internal and external ones, impinge on the behaving organism. Because traditional standardized measurement can properly take into account only the external forces acting on animate beings it is not adequate for measuring human behavior. Other approaches are called for if behavioral measurement is to lead to the establishment of lawful relations.

One such alternative is the idiographic filter which, as we have mentioned, focuses on the nature of relations both between manifest and latent variables and among latent variables but emphasizes invariant relations among the latter.

One may ask about alternatives to the idiographic filter as an approach to measurement. Obviously, classical test theory and item response theory will continue to be used, possibly with incremental changes in their nature to deal with particular concerns. Other possibilities have to do with adaptations of measurement invariance protocols such as partial invariance and configural invariance that offer some rationale for continuing more traditional latent variable modeling. More abstractly, mixed state-space modeling approaches (Chow, 2019) represent possible avenues to strengthening measurement. In this vein, non-linear extensions of the idiographic filter can be developed.

In science, there is an important balance in play between orthodoxy and novelty. This balance is evident in the behavior of grant reviewers and evaluators, journal editors, etc. Orthodoxy in science is a two-edged sword; it does a great service by resisting resource-consuming major efforts being made willy-nilly in many different directions but, at the same time, orthodoxy inhibits the introduction of novelty in conceptions that bear on the practice of science. It might be that one of the more important contributions of the idiographic filter notion involves “breaking out” of a deeply engrained orthodoxy involving the sanctity of traditional measurement invariance and its dependence on standardized measurement. Perhaps we’ll find that out. A striking example of orthodoxy’s two-edged nature can be seen in the way that measurement practice has managed to cling to the concept of measurement invariance in the context of the factor analytic model by “softening” the purer forms with notions such as configural invariance and partial invariance.

We believe that the study of behavior requires some modifications in the way we do science and have tried to identify some aspects of the situation. We have raised the possibility that for behavioral science, the traditional version of standardized measurement was too hastily adopted and cannot be relied on to accomplish for the measurement of behavioral attributes what it is purported to accomplish. If we have done this convincingly, the need for alternative measurement approaches is evident. The idiographic filter, which has the desirable properties of emphasizing the individual as the unit of analysis and explicitly focusing on invariant relations among general concepts without relying on standardized measurement protocols is one such alternative. It is our hope that others are forthcoming.

## Footnotes

1 If the nature of a process is general but its specific manifestation is different from one individual to another, sampling individuals in a representative way and synthesizing at the manifest variable level seems reckless. Because measurements are conducted on the observed variables, not the latent ones, the results of analyzing a representative sample’s standardized measurements yields an amalgamation that, while representing them statistically at the manifest level, may be meaningless at the latent level.

2 Perhaps it presses the point somewhat to regard A as a latent variable but, in fact, A is precisely defined by directly measurable variables such as length and width or radius. So, even though one may have the sense of being able to “observe” area, it is not directly measurable.

## Action editor

Lars-Gunnar Lundh served as action editor for this article.
